# Supplementary material for: MetaRibo-Seq measures translation in microbiomes
Source: Nat Commun. 2020 Jun 29;11:3268. doi: 10.1038/s41467-020-17081-z (PMC7324362; doi:10.1038/s41467-020-17081-z)
Supplement: Supplementary file 10 — Supplementary Data 7 [file 41467_2020_17081_MOESM10_ESM.zip › File2/Confidence_High_Taxonomy/107608_out.krona.html]

Javascript must be enabled to view this page.

members
magnitude
magnitudeUnassigned
count
unassigned
taxon
rank

107608\_out

8

2
superkingdom
8

8
phylum
1239


SRS098073\_contig\_number\_13707
1
species
1263000

7
186801
class


SRS146813\_contig\_number\_28349
1
order
7
186802

541000
family
3

216851
genus
1

853
species
1

SRS011084\_contig\_number\_21553

2

SRS015782\_contig\_number\_9221SRS049896\_contig\_number\_10437
species
1898205

1
family
186803

189330
genus
1

SRS014979\_contig\_number\_13875

species
1950906
1

SRS019496\_contig\_number\_contig-100\_26553.26554

1

SRS012849\_contig\_number\_24257
1898207
species
